# Cellular inhibitor of apoptosis-2 is a critical regulator of apoptosis in airway epithelial cells treated with asthma-related inflammatory cytokines

**DOI:** 10.1002/phy2.123

**Published:** 2013-10-11

**Authors:** Eugene Roscioli, Rhys Hamon, Richard E Ruffin, Susan Lester, Peter Zalewski

**Affiliations:** 1 Discipline of Medicine, The Queen Elizabeth Hospital, University of AdelaideWoodville, South Australia, 5011, Australia; 2 Rheumatology Unit, Queen Elizabeth HospitalWoodville, South Australia, 5011, Australia

**Keywords:** Apoptosis, asthma, epithelium, inflammation, inhibitor of apoptosis protein

## Abstract

Aberrant apoptosis of airway epithelial cells (AECs) is a disease contributing feature in the airways of asthmatics. The proinflammatory cytokines tumor necrosis factor α (TNFα) and interferon γ (IFNγ) are increased in asthma and have been shown to contribute to apoptosis at the airways. In the present study, we investigated the role of the inhibitor of apoptosis protein (IAP) family in primary AECs exposed to TNFα and IFNγ. IAPs are potent regulators of caspase activity elicited by the intrinsic and extrinsic apoptosis pathways. However, while caspase-mediated apoptosis was observed in AECs exposed to doxorubicin, it was not observed after cytokine treatment. Instead, AECs exhibited proapoptotic changes evidenced by an increased *Bax*:*Bcl2* transcript ratio and partial processing of procaspase-3. Examination by quantitative reverse transcription polymerase chain reaction (qRT-PCR) and Western analysis showed that proapoptotic changes were associated with a time- and dose-dependent induction of cellular IAP-2 (cIAP2), potentiated primarily by IFNγ. The abundance of the IAP antagonists X-linked IAP-associated factor 1 (XAF1) and second mitochondria-derived activator of caspases did not change, although a moderate nuclear redistribution was observed for XAF1, which was also observed for cIAP2. Small interfering RNA (siRNA)-mediated depletion of cIAP2 from AECs leads to caspase-3 activation and poly (ADP-ribose) polymerase cleavage, but this required extended cytokine exposure to produce a concomitant decrease in cIAP1 and Bcl2. These results indicate that AECs possess endogenous mechanisms making them highly resistant to apoptosis due to asthma-related inflammatory cytokines, and the activity of cIAP2 plays an important role in this protection.

## Introduction

The primary function of the airway epithelium (AE) is to maintain the airways for efficient ventilation. Central to this, the AE possess a remarkable regenerative capacity, with most airway epithelial cells (AECs) able to function as stem cells (Crystal et al. 2008). In asthma, the AE exhibits a fragile phenotype and inefficient repair after injury, a phenomenon hypothesized to sustain focused areas of AEC activation (Holgate 2011). Prolonged activation of AECs disrupts the epithelial barrier and leads to inappropriate secretion of immunomodulatory cytokines and growth factors (Holgate 2011; Lambrecht and Hammad 2012). The concept of a chronically injured epithelium has been convincingly linked with the unifying signs of asthma, such as Th2-related bronchial hyperreactivity, infiltration of inflammatory cells, and airway remodeling (Davies 2009; Bartemes and Kita 2012; Lambrecht and Hammad 2012). Consequently, dysregulation of the mechanisms regulating AEC apoptosis may significantly impact epithelial fragility and repair, and contribute to the disease.

The airways of asthmatics exhibit an elevated rate of epithelial apoptosis (Zhou et al. 2011), a phenomenon which increases with disease severity (Cohen et al. 2007). Conversely, infiltrating inflammatory cells are resistant to death in asthmatics (de Souza and Lindsay 2005), prolonging the release of factors such as transforming growth factor β (TGF-β), tumor necrosis factor α (TNFα), Fas ligand, and interleukin 1β (IL-1β), which can elicit apoptosis of AECs (Trautmann et al. 2002; Nakamura et al. 2004; Makinde et al. 2007; White 2011). However, apoptosis of AECs in asthmatics has been observed in the absence of prolonged inflammation, and demonstrate abnormalities for the production of Bcl2 and activation of caspases (Cohen et al. 2007; Holgate 2011; Zhou et al. 2011). Other factors shown to promote apoptosis of the AE include dysregulated zinc homeostasis (Roscioli et al. 2013), decreased production of E-cadherin (Trautmann et al. 2005), and heightened sensitivity to disease-related agents such as Fas ligand (White 2011). Whether elevated AEC apoptosis potentiates the fragile AE phenotype, or is a distinct phenomenon, remains unclear (White 2011). Further to this, less is known about the function of endogenous suppressors of the caspase cascade in the inflamed airways, and whether they exhibit deficits which may explain the aberrant apoptosis.

Members of the inhibitor of apoptosis protein (IAP) family are best known for their capacity to inhibit caspases; however, they also participate in other prosurvival activities (Roscioli et al. 2013). Of the IAPs, X-linked IAP (XIAP), cellular IAP-1 (cIAP1), and cIAP2, have been examined most rigorously due to their ubiquitous expression and association with cancer (Fulda and Vucic 2012). XIAP in particular is noted to inhibit caspase-3, -7, and -9, while some contention exists whether cIAP1 and cIAP2 inhibit caspases directly (Eckelman and Salvesen 2006). A more likely scenario is that multiple IAPs are required to maintain the apoptotic threshold (Moulin et al. 2012), and employ overlapping mechanisms to inhibit caspase activity. XIAP and the cIAPs have also gained significant attention through their involvement in a number of aspects of the immune response, including the regulation of the inflammasome and nuclear factor-kappa beta (NF-κB) signaling (Gyrd-Hansen and Meier 2010; Beug et al. 2012). Given the fragile nature of the AE in asthmatics, and the significant apoptotic pressure posed by the inflammation, dysfunction of the IAPs may have significant consequences for the integrity of the AE.

Here, we use primary AEC cultures stimulated with TNFα and interferon γ (IFNγ) to determine whether dysregulation of XIAP, cIAP1, and cIAP2 contributes to apoptosis observed in asthma-related inflammation. Although TNFα and IFNγ are pleiotropic cytokines which can influence several downstream pathways, their elevation in the airways of asthmatics has been shown to potentiate apoptosis of AECs (e.g., Trautmann et al. 2002, 2005). We hypothesize that apoptosis of AECs, at least in part, occurs through the reduction in IAP expression and function, or the upregulation of the IAP antagonists second mitochondrial-derived activator of caspases (Smac) and XIAP-associated factor 1 (XAF1).

## Experimental Procedures

### Human samples

Asthmatic (*n* = 10, five females, median age 50 years) and control subjects (*n* = 10, five females, median age 34 years) were selected from individuals attending clinics at the Queen Elizabeth Hospital and Lyell McEwin Hospital (Adelaide, Australia). Asthma status was based on self-report and previous diagnosis of asthma by a clinician. Asthmatic subjects exhibited mild-to-moderate, persistent form of the disease, and either did not require asthma medication, or used β2-receptor agonists (60%). Control volunteers were selected with no previous history of asthma and other respiratory diseases. Participants were free of conditions of the nasal cavity, and did not report a history of allergic rhinitis. This study was approved by The Queen Elizabeth Hospital and Lyell McEwin Hospital Ethics of Human Research Committee, and was conducted in accordance with the Declaration of Helsinki.

### Primary AEC culture

Informed consent was obtained prior to collection of AEC via nasal brushing. Nasal AECs were used as they are easily accessed, and do not require the donor to be sedated. In addition, nasal AECs exhibit comparable morphology to bronchial epithelial cells, and respond similarly in the context airway inflammation (McDougall et al. 2008). AECs were suspended in Bronchial Epithelial Growth Media (BEGM, Lonza, Walkersville, MD), and subject to monocyte depletion using anti-CD68 (Dako, Glostrup, Denmark) coated flasks for 20 min, in routine cell culture conditions (37°C, humidified, 5% CO_2_). Cells were expanded using type I collagen-coated flasks (Thermo Fisher, Waltham, MA), in BEGM. TNFα, IFNγ, and doxorubicin (all Sigma-Aldrich, St. Louis, MO) were used as treatment agents in AEC cultures. Cell cultures were confirmed to be of epithelial linage by professional cytologists (IMVS Cytology Department, The Queen Elizabeth Hospital, Adelaide, Australia), via reactivity to PAN-cytokeratin versus CD45 antibodies, and morphological examination via Diff-Quick analysis.

### Quantitative RT polymerase chain reaction

RNA was extracted from AEC cultures using the RNeasy RNA isolation method (Qiagen Valencia, CA). Complementary DNA (cDNA) was generated using MMLV reverse transcriptase (Invitrogen, Carlsbad, CA) and random hexamer RNA primers (Qiagen), and quantitative RT polymerase chain reaction (qRT-PCR) was performed using HotStar Taq polymerase kit (Qiagen) and the syto-9 DNA stain (Life Technologies, Carlsbad, CA). qRT-PCR was performed using the Corbett Rotor-Gene 6000 thermocycler (Qiagen), and results normalized to two endogenous control genes. Primers sequences, shown in Table 1, were designed in-house and synthesized by GeneWorks (Adelaide, Australia). All primer pairs were assessed for the production of a single PCR product, and reaction efficiencies exceeding 90%. Relative quantification was performed using the ΔΔCt method.

**Table 1:** Primers used for qRT-PCR

Gene name	Oligonucleotide sequences
*XIAP*	5′-CCGGCTGTCCTGGCGCGAAAA-3′ 5′-TTCCTTATTGATGTCTGCAGGTACACAAG-3′
*cIAP1*	5′-GATATCCTCATCTTCTTGAACAGCTGTTG-3′ 5′-TCCAGGTCCAAAATGAATAATTGGTGGG-3′
*cIAP2*	5′-TTACCCTCATCTACTTGAACAGCTGCTA-3′ 5′-TCTCCAGGTTCAAAATGGATAATTGATGAC-3′
*Smac*	5′-TCATAGGAGCCAGAGCTGAGATGAC-3′ 5′-CCTGATCTGCGCCAGTTTGATATGC-3′
*XAF1*	5′-GCCCAGCTCGGGAAAGGGGAAA-3′ 5′-CTGAGTCTGGACAACATTTACCCATATG-3′
*Bax*	5′-CAGTAACATGGAGCTGCAGAGGATGA-3′ 5′-ACCCGGCCCCAGTTGAAGTTGC-3′
*Bcl2*	5′-GTCATGTGTGTGGAGAGCGTCAAC-3′ 5′-AGTTCCACAAAGGCATCCCAGCC-3′
*Hypoxanthine phosphoribosyltransferase-1*^1^	5′-GGCTATAAATTCTTTGCTGACCTGCTG-3′ 5′-CAAAGTCTGCATTGTTTTGCCAGTGTC-3′
*Tata-binding protein*^1^	5′-CGAAACGCCGAATATAATCCCAAGCG-3′ 5′-CCAGTCTGGACTGTTCTTCACTCTTG-3′

1 Endogenous control gene.

### Western analysis

Protein was extracted from AEC cultures in situ, and Western blotting performed using the XCell SureLock Mini Cell System (Invitrogen). Membranes were probed with antibodies directed to β-actin (Sigma), Bcl2 (Santa Cruz, Dallas, TX), poly (ADP-ribose) polymerase (PARP), caspase-3, cIAP2 (both Cell Signaling, Danvers, MA), cIAP1 (R&D Systems, Minneapolis, NE), XAF1 (AbCAM, Cambridge, U.K.), and XIAP (BD Biosciences, San Diego, CA). Luminescence was detected using the LAS-4000 Imager (Fugifilm, Tokyo, Japan), and densitometry analyses performed using Multi-Gauge software (V3.0, Fugifilm Science Lab). Log-transformed density scores were normalized to both β-actin and the biological control.

### Fluorescent immunocytochemistry

AECs fixed in 2.5% neutral buffered formalin were simultaneously probed with anti-cIAP2 goat (R&D Sytems) and anti-XAF1 rabbit (Abcam) antibodies. Goat and rabbit mock immunoglobulin G (IgG) proteins were used to control for nonspecific binding of the respective primary antibodies. The nucleic acid stain 4′,6-diamidino-2-phenylindole (DAPI, Sigma) was used to resolve nuclei. Microscopy was performed using a Nikon Eclipse 90i microscope (Nikon, Tokyo, Japan).

### Apoptosis and necrosis assays

The processing of procaspase-3 and cleavage of PARP are events associated with apoptosis. Hence, western analysis antibodies were selected which detect both the unprocessed and cleaved forms of procaspase-3 and PARP. In addition, caspase-3/7 activity was detected using the FAM-FLICA fluorophore (ImmunoChemistry Technologies, Bloomington, MN) which binds to the catalytic site of these caspases. Microscopy was used to image fluorescence generate by caspase-bound fluorophores. Finally, qRT-PCR was used to determine modulation of *Bax* and *Bcl2* transcript levels. The relative abundance of Bax (potentiates cytochrome-*c* release from mitochondria) and Bcl2 (an inhibitor of cytochrome-*c* release) is an indicator of apoptotic changes (Salakou et al. 2007). Lactate dehydrogenase (LDH) released from compromised cells was used to quantify necrosis in cultures, according to the manufacturer's instructions (Cytotoxicity Detection Kit; Roche, Penzberg, Germany). In addition, cells grown in chamber slides were stained with propidium iodide to assess for necrotic cells, and Hoechst was used to resolve nuclei (both ImmunoChemistry Technologies).

### Small interfering RNA knockdown of cIAP2

Prevalidated small interfering RNA (siRNA) was used to deplete *cIAP2* transcripts in primary AEC cultures. cIAP2-specific siRNA (Qiagen, siRNA id: Hs_BIRC3_7) was transfected into AECs using HiPerFect transfection reagent (Qiagen). Scrambled siRNA oligonucleotide (AllStars Negative control siRNA; Qiagen) was used to control for nonspecific gene silencing as a result of the transfection process.

## Results

### Primary airway epithelia cells treated with TNFα and IFNγ demonstrate proapoptotic changes

**Figure 1:**
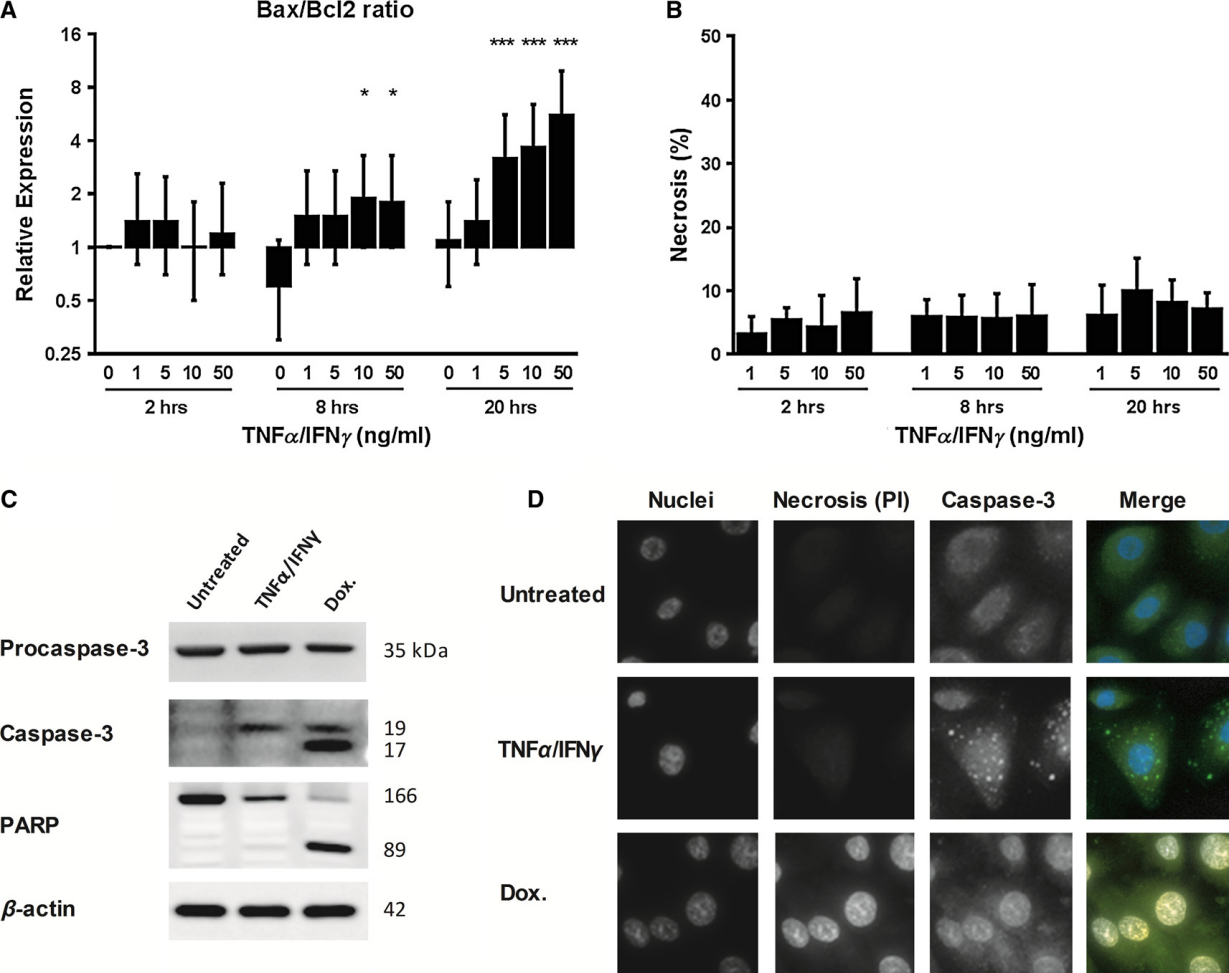
TNFα and IFNγ produce proapoptotic changes in primary airway epithelial cells (AECs). Cultured primary AECs from healthy donors were treated with TNFα and IFNγ, and assessed for apoptosis and necrosis. (A) Time- and dose-dependent elevation in the *Bax*/*Bcl2* transcript ratio is detected, particularly after 20 h of cytokine exposure (*n* = 5 donor AECs). Gene expression data were baselined to the 2-h untreated sample, and normalized to hypoxanthine phosphoribosyltransferase-1 and TATA box-binding protein reference genes. *P < 0.05; ***P < 0.001. Error bars represent 95% confidence intervals. (B) Cultures assessed for relative *Bax*/*Bcl2* transcript abundance were also examined for cytotoxicity, by measuring lactate dehydrogenase release into culture media. Error bars represent 95% confidence intervals. (C) Western analysis demonstrates procaspase-3 is partially processed in AECs treated with 50 ng/mL cytokines (single 19 kDa product). In contrast, doxorubicin (Dox; a control for caspase-mediated apoptosis) -treated cells (1 μmol/L for 20 h) exhibit complete procaspase-3 processing (both 19 and 17 kDa products), which is associated with cleavage of poly (ADP-ribose) polymerase (PARP). Results represent experiments using three AEC donors. (D) AECs treated with TNFα and IFNγ (50 ng/mL, 20 h) were assessed via immunofluorescence for necrosis using propidium iodide (PI), and caspase-3 localization. PI incorporation was not detected for AECs treated with proinflammatory cytokines, versus doxorubicin-treated (1 μmol/L) cells, which appear to exhibit secondary necrosis. Cells treated with proinflammatory cytokines demonstrate punctate regions of caspase-3 and/or -7 in the cytosol versus a more generalized and nuclear localization in doxorubicin-treated cells. Hoeschst staining was used to resolve cell nuclei. Results are representative of experiments performed using three AEC donors.

We first examined the apoptosis in primary AECs treated with TNFα and IFNγ. AEC from healthy donors treated with cytokines exhibit a time- and dose-dependent increase in *Bax*/*Bcl2* transcripts (Fig. 1A). Assessment of cytotoxicity in these cultures shows cell necrosis remained unchanged over the treatment regime (Fig. 1B). Supporting a proapoptotic outcome, cytokine treatment is sufficient to potentiate the generation of a 19 kDa caspase-3 subunit, however, the 17 kDa subunit was not detected (Fig. 1C). Consequently, PARP cleavage is not observed, as a second processing step needed to generate the caspase-3 heterodimer was inhibited or not elicited. Interestingly, AECs treated with cytokines exhibit punctuate regions of active caspase-3/7 within the cytoplasm, whereas doxorubicin produces a nuclear and cytosolic distribution (Fig. 1D). In agreement with results for cytotoxicity, propidium iodide is excluded from AECs treated with the cytokines. These results indicate primary AEC resist apoptotic stimuli brought about by TNFα and IFNγ, and may employ endogenous mechanisms to arrest the caspase cascade.

### TNFα and IFNγ elevate *cIAP2* and *XAF1* transcription in AECs from asthmatic and control donors

**Figure 2:**
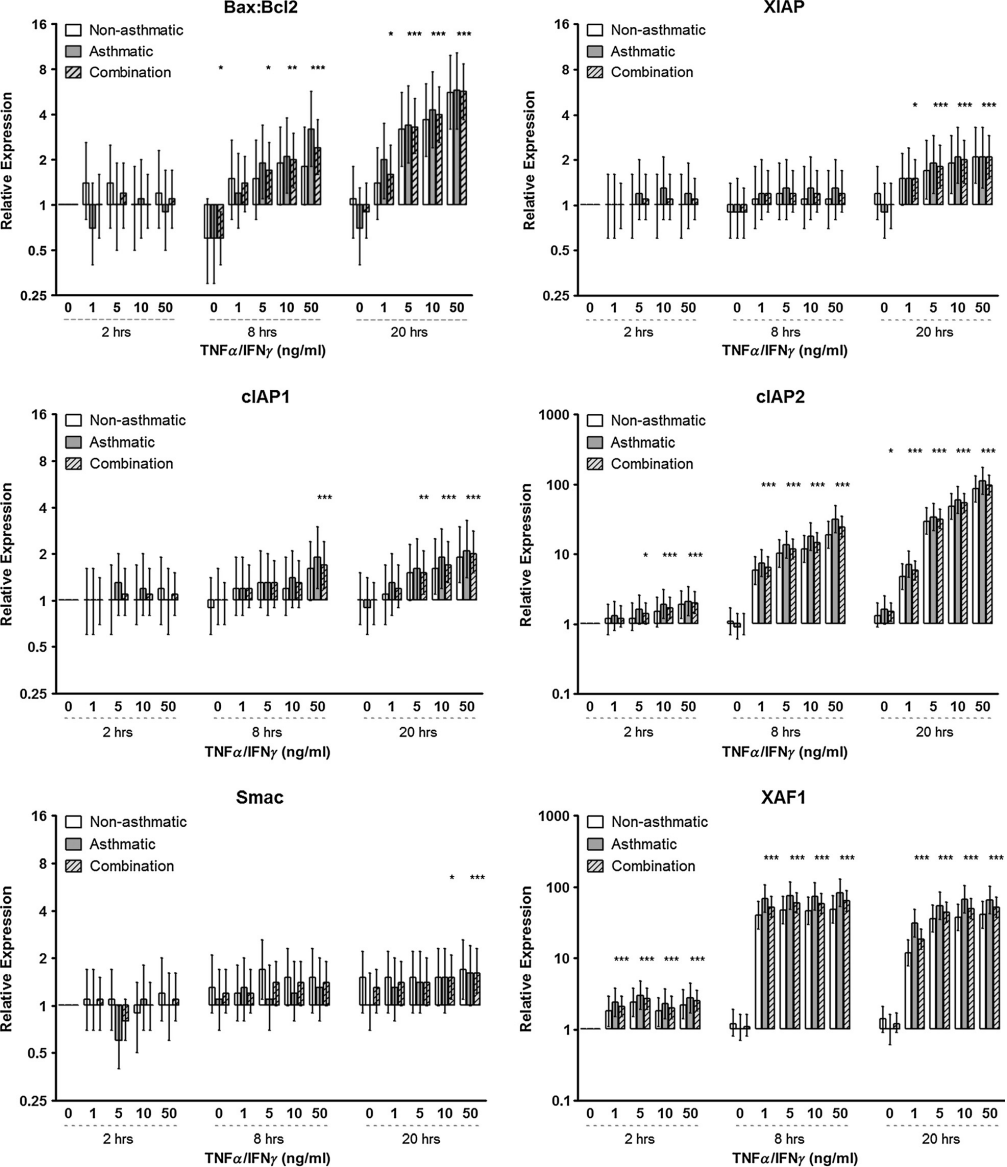
Airway epithelial cells (AECs) from asthmatic and healthy donors exposed to proinflammatory cytokines, exhibit corresponding levels of inhibitor of apoptosis protein (IAP) transcript abundance. Cultured primary AECs from asthmatic (*n* = 5) and nonasthmatic (*n* = 5) donors were cultured in the presence of TNFα and IFNγ, and assessed for *Bax*/*Bcl2*, IAP, and IAP-antagonists transcript abundance. Gene expression data were baselined to the 2-h untreated sample, and normalized to hypoxanthine phosphoribosyltransferase-1 and TATA box-binding protein reference genes. *P < 0.05, **P < 0.01, and ***P < 0.001, and pertain to the combined expression data. Error bars represent 95% confidence intervals.

Next, we determined whether the expression of cIAP1, cIAP2, and XIAP, or their antagonists XAF1 and Smac, modulate in a manner consistent with resistance to cytokine-induced apoptosis. The increased *Bax:Bcl2* transcript ratio previously observed in nonasthmatics is similarly elevated in AECs from asthmatic donors (Fig. 2). In line with this observation, the expression of each transcript was found to be comparable between AECs derived from control and asthmatic participants. Although, XIAP, cIAP1, and Smac demonstrate marginal elevation in gene expression after 20-h cytokine treatment, cIAP2 exhibited a considerably time- and dose-dependent increase. Interestingly, an escalation in XAF1 transcription may represent a response to counter increasing levels of cIAP2.

### Induction of cIAP2 by inflammatory cytokines is associated with partial processing of procaspase-3

**Figure 3:**
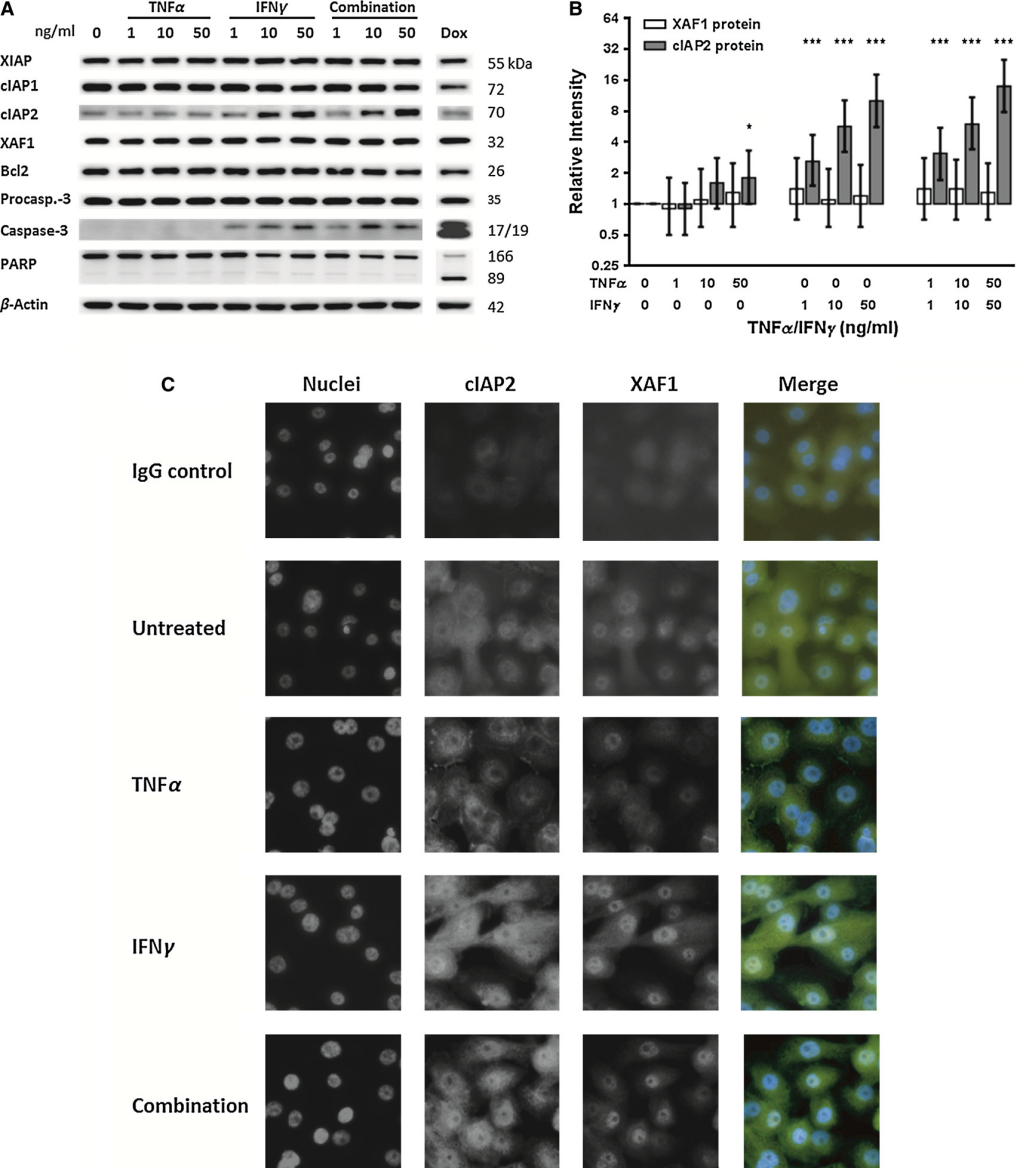
Cellular inhibitor of apoptosis-2 protein is upregulated in response to IFNγ in primary airway epithelial cells (AECs). Cultured primary AECs from nonasthmatic donors (*n* = 3) were treated with TNFα and/or IFNγ for 20 h, and protein expression assessed via Western analysis and immunocytochemistry. (A) An increase in cIAP2 protein abundance in AECs treated with IFNγ correlates with the production of a single 19 kDa cleaved caspase-3 band, but not cleavage of poly (ADP-ribose) polymerase (PARP). Doxorubicin (Dox) is used as a positive control for caspase-mediated apoptosis. (B) Densitometry analyses of Western blot results confirm a dose- and time-dependent increase in cIAP2 protein abundance, particularly in response to IFNγ. In contrast, XAF1 protein does not alter with cytokine treatment. Protein expression was baselined to the abundance in the untreated sample, and normalized to the expression of β-actin. *P < 0.05, **P < 0.01, and ***P < 0.001, and pertain to the cIAP2 expression data. Error bars represent 95% confidence intervals. (C) XAF1 and cIAP2 have a uniform distribution throughout the cell, with a moderate nuclear relocalization in response to IFNγ. IgG cont. refers to an isotype control antibody. 4′,6-diamidino-2-phenylindole (DAPI) was used to resolve cell nuclei.

We next determined whether IAP protein abundance reflects changes observed in gene expression. As for its transcript, XIAP protein expression remained stable over the treatment regimen (Fig. 3A). cIAP1 protein also remain consistent, although a nonsignificant reduction with both cytokines (2.75-fold, *P* = 0.062) was observed. Basal levels of cIAP2 were often undetected in untreated AEC. However, exposure to IFNγ or both cytokines lead to a dose- and time-dependent induction in cIAP2, matching XIAP and cIAP1 (Fig. 3A and B). Interestingly, induction of cIAP2 coincides with the generation of a single 19 kDa caspase-3 product, which is for the most part potentiated by IFNγ. Conversely, the previously observed increase in *XAF1* transcript did not produce an associated elevation in protein abundance. Bcl2 protein levels also remained stable, with the exception of a nonsignificant reduction with 50 ng/mL of both cytokines (1.5-fold, *P* = 0.19). Immunofluorescent analysis shows that XAF1 and cIAP2 share a uniform distribution throughout the cytosol, and a moderate increase in nuclear localization as a result of IFNγ and combination treatment (Fig. 3C).

### Doxorubicin inhibits cytokine-mediated cIAP2 induction, and is associated with apoptosis of AECs

**Figure 4:**
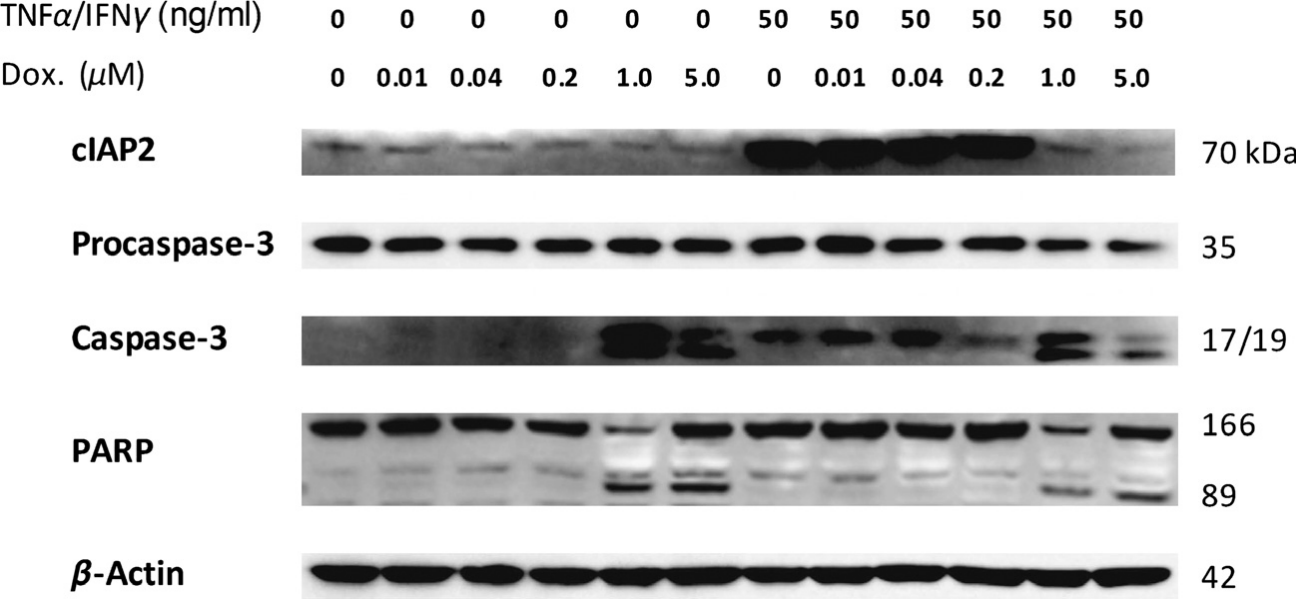
Doxorubicin-induced downregulation of cIAP2 correlates with airway epithelial cells (AECs) apoptosis. Cultured primary AECs were treated for 20 h with increasing concentrations of doxorubicin (Dox), in the presence or absence of 50 ng/mL TNFα and IFNγ. TNFα and IFNγ potentiate the induction of cIAP2 until doxorubicin concentration reaches 1 μmol/L, after which cIAP2 expression is inhibited and apoptosis is detected. Results are representative of experiments using three AEC donors.

While using doxorubicin to provide a positive control for caspase-mediated apoptosis, we observed that a reduction in cIAP1 and Bcl2 protein expression was consistent with apoptosis (e.g., Fig 3A). Hence, to determine whether doxorubicin also arrests cIAP2 induction, AECs were treated with doxorubicin in the presence or absence of TNFα and IFNγ. Approximately 1 μmol/L of doxorubicin induces apoptosis in AEC after 20 h (Fig. 4). Interestingly, this concentration also inhibits cytokine-mediated cIAP2 induction, reducing its protein abundance to basal levels. Inhibition of cIAP2 correlates with complete procaspase-3 processing, and apoptosis as evidence by PARP cleavage. Of note, the proapoptotic influence of TNFα and IFNγ did not sensitize AECs to apoptosis using doxorubicin below 1 μmol/L, where cIAP2 expression remains elevated. Consequently, basal level cIAP2 with concomitant reduction in cIAP1 and Bcl2 may produce conditions suitable for apoptosis in AECs.

### Downregulation of cIAP1 and Bcl2 is associated with cytokine-induced apoptosis in cIAP2-depleted AECs

**Figure 5:**
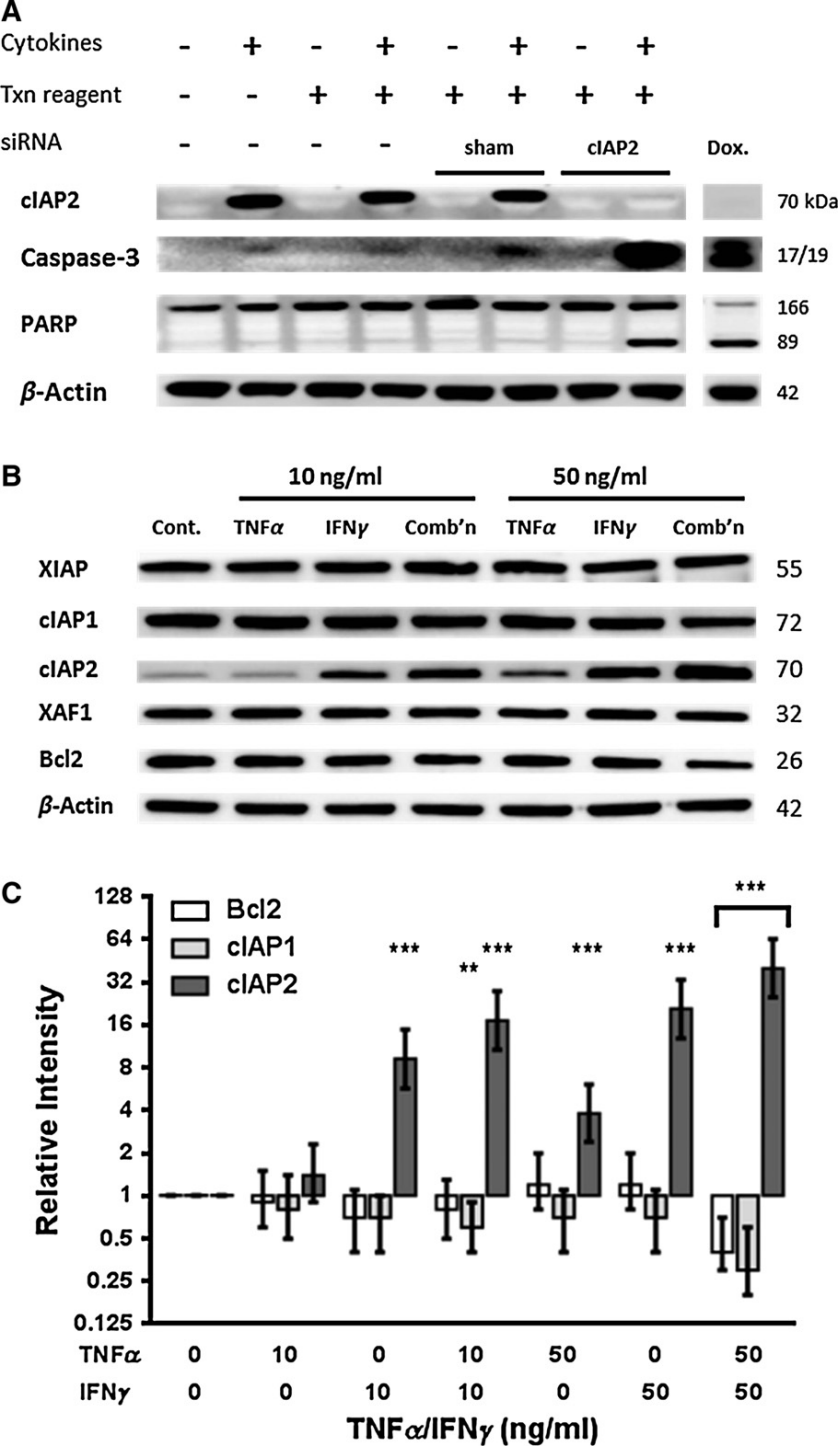
Apoptosis of cIAP2-depleted airway epithelial cells (AECs) by TNFα and IFNγ correlates with cIAP1 and Bcl2 downregulation. (A) AECs from nonasthmatic donors were transfected with cIAP2-specific siRNA, and cultured for 72 h in the presence of TNFα and IFNγ (50 ng/mL). A large 19 kDa caspase-3 product is produced, with a relatively small 17 kDa product. Transfection (Txn) reagent and scrambled siRNA (sham) oligonucleotides were included to control for unintended cIAP2 silencing, and apoptosis as a result of transfection conditions. Doxorubicin (Dox) was used as a positive control for caspase-mediated apoptosis. These results are representative of experiments performed using three AEC donors. (B) Primary AECs treated with TNFα and IFNγ for 72 h exhibit downregulation of cIAP1 and Bcl2 protein abundance, while XIAP and XAF1 levels remain stable. (C) Densitometry analysis of Western blots (*n* = 3) shows cIAP1 and Bcl2 downregulation becomes significant when AECs are treated with 50 ng/mL of TNFα and IFNγ. Results are representative of three experiments using different AEC donors. Protein expression was baselined to the abundance in the untreated sample, and normalized to the expression of β-actin. **P < 0.01; ***P < 0.001. Error bars represent 95% confidence intervals.

As cIAP2 upregulation by TNFα and IFNγ correlates with AEC survival, and this induction is inhibited by doxorubicin, may indicate cIAP2 has a role in preventing apoptosis. To examine this, siRNA was used to knockout *cIAP2* transcripts in AECs stimulated with cytokines. AECs depleted of cIAP2 and treated for 20 or 48 h exhibited the previously observed pattern of incomplete procaspase-3 processing, and full-length PARP (not shown). However, extending cytokine exposure to 72 h potentiated conditions permissive to caspase-3 activation and PARP cleavage (Fig. 5A). In the absence of cIAP2, procaspase-3 processing favors the generation of the 19 kDa caspase-3 subunit. This may have limited the formation of caspase-3 heterodimers, as incomplete cleavage of PARP was a consistent observation. Unlike earlier time points, extending cytokine exposure to 72 h caused a significant reduction in cIAP1 and Bcl2 protein abundance (Fig 5B and C). This suggests that apoptosis as a result of cIAP2 depletion may depend on reductions in cIAP1 and Bcl2, which is in agreement with finding for doxorubicin-induced AEC apoptosis.

## Discussion

In this study, we report that AECs exhibit a proapoptotic phenotype when exposed to asthma-related proinflammatory cytokines, and cIAP2 in partnership with cIAP1 and Bcl2, may be primary factors blocking commitment to apoptosis. In comparison to other reports (e.g., Trautmann et al. 2002, 2005; Zheng et al. 2005; Stout et al. 2007; Basinski et al. 2009), we were unable to elicit apoptosis exclusively through exposure to TNFα and IFNγ, a situation which may reflect differences in cell culture (White 2011). For example, unlike AECs cultured directly from donors, the commonly used normal human bronchial epithelial (NHBE) cell line can grow on a plastic substratum, and are often examined at subconfluence. Primary cells are more resistant to cell death, particularly on surfaces such as collagen at densities over 85% (Aoshiba et al. 1997; Shi et al. 2002; Leverkus et al. 2003). Hence, NHBE shedding often ascribed to TNFα- and IFNγ-related apoptosis may mask other important cell survival mechanisms. In line with this, recently Liao et al. (2011) found that NHBE cells are resistant to IFNγ, and targeted downregulation of apolipoprotein was required to produce caspase-dependent apoptosis. Here, AECs demonstrated proapoptotic changes, evidence by an increased *Bax*:*Bcl2* transcript ratio, and partially processed procaspase-3 in the absence of PARP cleavage (Fig. 1A and C). Interestingly, immunofluorescence revealed localized regions of caspase activation within the cytosol of cytokine-treated cells (Fig. 1D). As active caspases were not detected during western analysis, this observation may represent caspases incorporated into protein complexes, or an artifact of the fluorophore associating with partly processed procaspases. The presence of partially cleaved procaspases without progression into apoptosis indicates regulation by the IAPs.

The significance of IAP expression in the inflamed airway is yet to be explored. We found that basal levels of XIAP and cIAP1 were high in AEC, implying a sentinel function. Conversely, basal level cIAP2 was often below detection, but was strongly induced by IFNγ (Fig. 3A). This suggests that the cIAPs are differentially regulated, and cIAP2 may provide a cytoprotective function. However, investigations using the A549 AEC line demonstrated that IFNγ did not influence IAP expression, and inhibited TNF-related apoptosis-inducing ligand-mediated upregulation of cIAP2 (Wen et al. 1997; Park et al. 2002, 2004). Interestingly, similar IAP expression is found in neutrophils stimulated with IFNγ, which demonstrate a moderate upregulation in cIAP2 via activation of the JAK-STAT pathway (Sakamoto et al. 2005). In addition, the cIAP2 promoter contains a putative binding element for interferon regulatory transcription factor 1, which is known to regulate several proteins involved in apoptosis (Hong et al. 2000; Maher et al. 2007). Rat hepatocytes treated with a TNFα and IFNγ also upregulate cIAP2, but likely as a result of TNFα/NFkB signaling (Schoemaker et al. 2002; Peng et al. 2012). Although the cIAP2 promoter contains NF-κB-responsive elements (e.g., Jin et al. 2009), TNFα potentiated a modest increase in cIAP2 in AECs examined here. Of the IAP antagonists, a vast increase in XAF1 transcription did not translate into elevated protein abundance, suggesting influential posttranslational regulation. However, XAF1 and cIAP2 were observed to exhibit a partial relocalization from the cytosol to the nucleus after stimulation with IFNγ (Fig. 3D). XAF1 is known to complex with XIAP and sequester it to the nucleus (Liston et al. 2001). cIAP2 can also localize to the nucleus (Mekhail et al. 2007) and is known to bind XAF1 (Arora et al. 2007), but whether this occurs as a proapoptotic mechanism through XAF1–cIAP2 complexes requires further investigation. We were unable to detect altered gene expression in AECs derived from asthmatics versus controls (Fig. 2), which may suggest there is no intrinsic defect in IAP regulation.

The cIAP2 induction correlated with partial processing of procaspase-3, pointed toward cIAP2 preventing progression into apoptosis. During our use of doxorubicin, we found that downregulation of cIAP1 and Bcl2, with basal level cIAP2, was consistent with apoptosis (e.g., Fig. 3A). Further to this, induction of cIAP2 by proinflammatory cytokines was abolished by doxorubicin (Fig. 4), suggesting that basal levels of cIAP2 may be a requirement for caspase activation in primary AECs. Similarly, targeted depletion of cIAP2 using siRNA did not result in caspase-3 activation, unless cytokine exposure was extended to produce a significant reduction in cIAP1 and Bcl2 (Fig. 5). This may mean cIAP2 plays a distinct prosurvival role that becomes apparent after the overlapping activities of cIAP1 and Bcl2 decline. These findings are consistent with other reports which found apoptosis through reduced IAP surveillance requires the depletion of more than one IAP (Rumble et al. 2008; Moulin et al. 2012). Sensitization to apoptosis by depleting the cIAPs, without a reduction in XIAP, has been observed in a number of investigations (e.g., Ruemmele et al. 2002; Kim et al. 2012). Here, cIAP2 knockout strongly favored the generation of a single caspase-3 product (Fig. 5A), suggesting that cIAP2 regulates the initial step of procaspases-3 maturation, performed by initiator caspases. The imbalance in caspase-3 products may have limited the formation of active caspase-3 heterodimers. However, XIAP is a potent inhibitor of caspase-3 enzymatic activity, and can therefore inhibit activated caspases which perform the final cleavage for procaspases-3 maturation (Zorn et al. 2012). Hence, abundant XIAP in cytokine-treated AECs was likely responsible for the imbalance in procaspases-3 processing observed after cIAP2 knockdown. Indeed, convincing evidence exists that the cIAPs do not possess the necessary residues to physically interact with caspases in vivo (Eckelman and Salvesen 2006), and they primarily effect cell survival through alternative mechanisms (summarized in Roscioli et al. 2013). Hence, cIAP2 is no doubt an influential prosurvival factor, but may not be sufficient to protect against apoptosis in isolation.

Here, AECs from asthmatic and control donors produced similar IAP expression profiles when treated with proinflammatory cytokines. Given the fragile nature of AECs derived from asthmatics, we hypothesized that IAP expression may be downregulated after exposure to TNFα and IFNγ. The use of air–liquid interface culture models have been shown to emphasize asthma-associated phenotypes exhibited by AECs during in vitro investigations (e.g., Hackett et al. 2011, 2013). Consequently, we are now examining the possibility of whether an air–liquid culture system will define differences in IAP expression, in AECs derived from asthmatics.

Our findings demonstrate that cIAP2 is a critical factor providing resistance to AECs exposed to asthma-related inflammation. Contemporary asthma therapies which passage the AE to target the mediators of inflammation and bronchial constriction overlook disease-causing factors presented by AECs. Further to this, there is strong evidence that some asthma medications contribute to AE apoptosis (Dorscheid et al. 2006). In the future, therapeutics with the duel objective of preventing inflammation and maintaining the viability of the AE may provide significant progress toward countering the perpetuating factors associated with asthma.
